# General Clinic Atlanta Medical College

**Published:** 1873-01

**Authors:** 


### GENERAL CLINIC ATLANTA MEDICAL COLLEGE.

SERVICE OF WM. ABRAM LOVE, M. D.,

*Professor of Physiology and Clinical Lecturer on Diseases of the Liver and Kidneys.*

CASE 5. *Rheumatism, Acute, with Intermittent Fever.*—J. M., æt. forty, colored, and a widow, an in-door laborer, was taken a week ago with a chill, followed by fever passing off with diaphoresis; at the same time she suffered from pain in the right knee and ankle joint, with heat, swelling, and an inability to move them without much suffering. Slight chills, followed by fever, have returned on alternate days, until the present. She ceased menstruating in May last; has suffered no inconvenience in this respect; tongue slightly coated; fauces somewhat discolored—of a yellowish tinge; bowels regular; urine acid; heart sounds normal; respiration good; and she suffers only with the chills and fevers, and pain in the knee and ankle. The joints, as you will observe, are very much swollen. She has been the subject of rheumatism before, the evidence of which is presented in the enlargement and stiffness of some of the finger joints of the right hand, as you see. We have, then, in this case to contend with rheumatism complicated with malarial poison; they are not at all incompatible with each other, nor is the treatment; we shall, therefore, recommend—

℞.—Quinia sulph., grs. vi.

Morphia sulph., gr. $\frac{1}{4}$, *ter die*—

To arrest the malarial fever and allay pain, a cholagogue cathartic (i.e., pil. cath. comp. U. S. P.) to evacuate the bowels and remove portal engorgement; to be followed by—

℞.—Pot. bicarb., grs. xxx, *ter die*—

Largely diluted with water, as an eliminant and ant-acid, to correct any acidity that may exist in the blood. The quinine to be continued until the fevers are arrested; the cathartic to be repeated as occasion may require to keep down portal engorgement and the bowels open; while the alkali, increased if necessary, will be persisted in until the rheumatic symptoms have subsided, unless it shall too much reduce the system; in that event it should be alternated with quinine, or the bitter tonics and iron, or omitted for a few days. The urine will furnish an index to the blood, and the alkali should be pushed while this shows an acid reaction with litmus paper.

CASE 6. *Acute Rheumatism with Chills and Fever.*—N. P., æt. forty-eight, colored, carpenter; has been subject to chills and fever occasionally for two or three years. Last winter and fall they returned on him every few weeks, after being arrested with quinine and other remedies. He has had them occasionally this fall. With his present attack he suffers also with pain in the right knee; this as you see is swollen, and any movement of the joint is accompanied with pain. Bowels constipated; tongue coated with a brown fur; soft palate of a golden hue; pulse febrile—about 100 per minute; respiration good; heart sounds normal; kidneys acting well, but urine acid and highly colored; appetite rather fastidious—food often disagreeing with the stomach.

℞.—Hydrarg. chlor. mit., grs. iij.
Pv. ipecac et opii co., grs. v.

At one, four, seven and ten o'clock, P.M., to be followed next morning with—

℞.—Ol. Ricini, f ℥ i.

Ol. terebinth., gtt. xx.

And repeated if necessary.

*Second day.*—Cholagogue and cathartic produced copious bilious discharges, with some relief to patient's feelings, but pain continues in knee.

℞.—Quinia sulph., grs. v.
Pv. ipecac et opii co., grs. v.
Pv. camph. gr. i, *ter die.*

℞.—Potas. bicarb., grs. xxv, in aq. f ℥ viij, *ter die.*

℞.—Linamentum terebinth, to be applied to knee.

Treatment to be continued; bowels to be kept open; diet, fluid, but nutritious; stimulants avoided; until recovery.

*Remarks.*—These cases present to you, gentlemen, fair specimens of *articular rheumatism*, located; and as we have a few moments not otherwise appropriated, you will excuse me, I know, if that time is devoted to the subject of acute rheumatism and its treatment. In these cases, here present, there is no difficulty in diagnosis; the disease has presented itself unmistakably in the joints. In other cases you will find it as it were, afloat, and more difficult to recognize—pains of a rheumatic character presenting themselves here and there in the articulations, or attacking the bursæ of the muscles; resting now in one joint, with more or less heat, pain and swelling, accompanied with general febrile disturbance, then as suddenly disappearing, to reappear in some more or less distant part; nor is this confined to the articulations: the muscular tissue, the heart, stomach, the intestinal canal, it would seem, are all subject to attacks in some cases where a rheumatic diathesis exists, though they may pass under different names, when we consider the symptoms alone without reference to the peculiar conditions of the system existing in rheumatism. Females who are the subjects of the disease will often suffer with attacks affecting the uterine muscles; and during gestation it may lead to premature labor, and I believe often does, where the true cause has not been discovered and removed by proper treatment. But in all cases, more especially in those of an active inflammatory character, with a migratory disposition, the great danger is the development of cardiac disease, carditis or pericarditis, the deposits of fibrin on the cardiac valves, as also, I believe, in membranous croup, diptheritic affections, etc. Arthritic deposits are less serious, though these too are to be deprecated. But even in this class of cases the cardiac complications may come up as well and prove immediately or remotely serious in their character. I allude to these for the purpose of warning you in the treatment to use your utmost endeavor to prevent these complications. Pass no case of suspected rheumatism without strictly scrutinizing the cardiac condition, and reckon no case relieved where abnormal sounds mark the effects of the disease.

The multifarious plans of treatment recommended, the great number of remedies used from time to time, but serve to show the formidable character, and in many instances, unyielding nature of, this affection. The old plans of treatment were, in the calomel era, mercurials to ptyalism, in the bloody age, *venesectio ad deliquium animi;* then followed contra stimulants: water cures—hot and cold, blisters, quinine, opium, nitrate of potash, lemon juice, etc., etc.; and lastly, what is now known as the alkaline treatment. All these plans have had their advocates, and doubtless have them still; while rational medicine, based on our present knowledge of pathology and therapeutics, would seem to point out neither the one nor the other, exclusively, but a combination dictated by the indications in each and every individual case.

It is conceded by most medical men of the present day, that the peculiar diathesis or condition of the system giving rise to the disease, is that of undue acidity of the blood—lithic or lactic—together with increase of fibrin in that fluid, particularly in the sthenic and acute forms of the disease. This pathological condition would naturally point to alkalies, and especially to alkaline carbonates, as the proper remedies to neutralize this super acidity—to remove the cause—and the results of experience prove the correctness of these conclusions. Both in this country and in Europe, this plan has proved most successful; and in cases thus treated there have occurred fewer cardiac complications. In this we better understand the effects of the "lemon juice treatment," though an acid and apparently contradictory. If we remember that the citrates, tartrates and malates are soon converted into carbonates in the system, this seeming contradiction, is explained.

In the treatment the alkalies and alkaline carbonates have been, it would appear, used rather indiscriminately: soda, potash, ammonia, lithia, etc, I have elsewhere endeavored to explain to you the difference in the action of the soda and the potash salts—the assimilative action of the one and the eliminative action of the other, as demonstrated by Woronochin and Zebelen—and for this reason, as an eliminative alkali the potash salts are to be preferred in the treatment of this and other diseases, where it is desired to reduce the blood tissue. But in its use the remedy must not be carried too far, especially in cases of an asthenic character, particularly where there exists at the same time a tendency to scrofula. Their power of thinning the blood is very great, and by their long continued use, that tissue may be so broken down that you will find it difficult to rebuild. It is better in such cases to suspend the remedy for a short time and give tonics, or mix, or alternate with the soda salts, with or without iron, quinine, etc.

In this malarial region of country you will seldom find quinine amiss in the treatment of rheumatism, and I have been long in the habit of alternating it with the eliminative alkalies, not only as a malarial antidote, but as a tonic, to aid in the toning of the cell walls—in the rebuilding of tissue, so to speak. I believe that it is in this way quinine has acted in the hands of those who have trusted to what is called the "quinine treatment" of rheumatism.

In some cases I have seen the happiest results from the use of colchicum, especially in alternation with the alkalies; but I must say in others I have found no good follow its administration, under apparently the same condition and surroundings. I could not attribute this difference to a difference in the drug, for I had used the same in both cases. Another, and an indigenous medicine, to which I will ask your favorable consideration, is the cimicifuga racemosa, actæa. This, like the one just mentioned, in many cases acts most beneficially, especially in controlling rheumatic fever. Thirty to fifty drops of the concentrated tincture every two to four hours has a happy effect in reducing the fever, particularly in the early stages of the attack. I have given it combined, or alternated with the alkalies, with the most marked improvement to the condition of my patients. How it acts I can no more tell you than I can how it acts in chorea, epilepsy and hypochondriasis. It does not seem to be an eliminative in any sense, but an antidote in some way to the rheumatic poison; whether through the blood or through the nerve system must be passed for the future to settle.

You will find in your patients suffering from this painful disease, that as night-fall approaches they will appeal to you for something to give them rest for the night. I am pursuaded that you will find no combination more effectual in securing this boon than a combination of bromide of potassium, opium and ipecac. I have been in the habit of substituting the bromide for the sulphate of potass, as in Dover's powders, but in double the quantity and giving it in double the dose. I think you will be pleased with the results when in the future you may have occasion to resort to the combination, not for the purpose of producing nocturnal rest alone, but for the relief of pain at any period in the treatment. Blisters near the affected joints you will find too, gentlemen, to give much relief and aid in promoting recovery. Many physicians here and abroad place their main reliance on blisters; and repeated blistering, used one after another, until the blistered surface in some cases amount to two, three, four, or even six hundred square inches, in the treatment of a case. Should you deem it advisable to resort to the use of this remedy, my advise to you is, do not add the pain of strangury to the pangs of rheumatism, but prevent it by the proper preparation of your plasters. A few grains of powdered opium, or an equivalent of morphine, with powdered camphor, thinly spread over your plaster before applying it, will not only prevent strangury, but will very greatly allay the burning pain while vesication is taking place. By repeated blistering you will correct the acidity of the blood and reduce the system as with alkalies. You may find it expedient to double, or treble, or quadruple your remedies in some severe cases, using alkalies, blisters, quinine, actæa, etc.; but as I have told you before, you must watch your patients and see to it that they are not too far reduced. While you reduce the fibrin of the blood and remove acidity, you may find iron requisite to give vigor to its corpuscular portion; if so, do not hesitate to give it, more especially in cases with rapid tendency to an asthenic or anæmic condition.

Of hypodermic medication, of linaments and other local applications, I have not now time to speak, nor have I of the action of the nitrate of potash internally administered or externally applied.

The necessity for a fluid diet, the avoidance of stimulants of any kind, and absolute rest, in the more active forms and the more acute stages, will be sufficiently evident, while attention to the secretions and excretions, to proper nutrition and to any complications arising or threatened, must be left to your own good judgment in the progress of each and every individual case.

